# CircRNAs in osteoarthritis: research status and prospect

**DOI:** 10.3389/fgene.2023.1173812

**Published:** 2023-05-09

**Authors:** Zhuang Li, Jun Lu

**Affiliations:** ^1^ School of Medicine, Southeast University, Nanjing, Jiangsu, China; ^2^Department of Orthopaedics, Zhongda Hospital, School of Medicine, Southeast University, Nanjing, Jiangsu, China

**Keywords:** circRNA, osteoarthritis, cartilage, molecular mechanism, synovium

## Abstract

Osteoarthritis (OA) is the most common joint disease globally, and its progression is irreversible. The mechanism of osteoarthritis is not fully understood. Research on the molecular biological mechanism of OA is deepening, among which epigenetics, especially noncoding RNA, is an emerging hotspot. CircRNA is a unique circular noncoding RNA not degraded by RNase R, so it is a possible clinical target and biomarker. Many studies have found that circRNAs play an essential role in the progression of OA, including extracellular matrix metabolism, autophagy, apoptosis, the proliferation of chondrocytes, inflammation, oxidative stress, cartilage development, and chondrogenic differentiation. Differential expression of circRNAs was also observed in the synovium and subchondral bone in the OA joint. In terms of mechanism, existing studies have mainly found that circRNA adsorbs miRNA through the ceRNA mechanism, and a few studies have found that circRNA can serve as a scaffold for protein reactions. In terms of clinical transformation, circRNAs are considered promising biomarkers, but no large cohort has tested their diagnostic value. Meanwhile, some studies have used circRNAs loaded in extracellular vesicles for OA precision medicine. However, there are still many problems to be solved in the research, such as the role of circRNA in different OA stages or OA subtypes, the construction of animal models of circRNA knockout, and more research on the mechanism of circRNA. In general, circRNAs have a regulatory role in OA and have particular clinical potential, but further studies are needed in the future.

## 1 Introduction

Osteoarthritis (OA) is a classic degenerative chronic disease with significant symptoms, including pain, morning stiffness, and joint instability, leading to disability and ultimately impairing quality of life (Martel-Pelletier et al., 2016). The incidence of osteoarthritis is still high worldwide, with approximately 303.1 million hip and knee osteoarthritis cases, according to the Global Burden of Disease Project (GBD). As presented by Safari et al.'s analysis of GBD data up to 2017, the incidence of osteoarthritis has increased by approximately 8%–10% since 1990, which is based only on hip and knee osteoarthritis (Peat and Thomas, 2021). As a chronic disease, osteoarthritis impacts patients’ quality of life and burdens the country and society long-term. Although, at present, the subtypes of OA, risk factors, or etiologic factors, the mechanism of development cannot be revealed entirely clearly. The corresponding treatment methods are also under study. Osteoarthritis is characterized by cartilage degeneration, osteophyte formation, damage and remodeling of the cartilage, and varying degrees of synovitis and other joint structural damage, including ligaments and menisci. Identifying the molecular biological mechanisms of osteoarthritis development is essential to the treatment of osteoarthritis (Katz et al., 2021). The current mechanism of osteoarthritis development mainly focuses on changes in the structure of tissues and their functions in the joints, with the participation of various molecular biological processes and a variety of cells and cytokines (van den Bosch, 2021). The understanding of the molecular biological mechanism is constantly improving, including epigenetics as a new hot research direction in recent years. The osteoarthritis research process is increasingly often mentioned, of which noncoding RNA research is also increasingly in-depth, especially circRNA research (Ratneswaran and Kapoor, 2021). Osteoarthritis is an inflammatory disease produced by various factors, and circRNAs also participate in and regulate its progression (Zhang et al., 2021a). Circular RNA is currently one of the heated topics in research, and the purpose of this paper is to review the progress of its research in osteoarthritis and discuss its significance, breakthroughs, and deficiencies in current research and future research directions.

## 2 Introduction to CircRNA

A wide variety of noncoding RNAs are involved in osteoarthritis, including microRNAs, lncRNAs, and circRNAs. CircRNA is a long-chained and closed-loop RNA with better stability and a longer half-life due to its unique circular structure, which is more resistant to RNase R (Jeck and Sharpless, 2014), making it a potential candidate for diagnostic biomarkers and therapeutic targets. CircRNAs form by reverse splicing, wherein the 3′end of the exon is connected to its own or upstream exon’s 5′end through a 3'- 5′phosphate bond, forming a closed-loop structure with a reverse splicing connection site (Chen and Yang, 2015). They participate in a variety of physiological or pathological processes through a variety of mechanisms.

**FIGURE 1:**
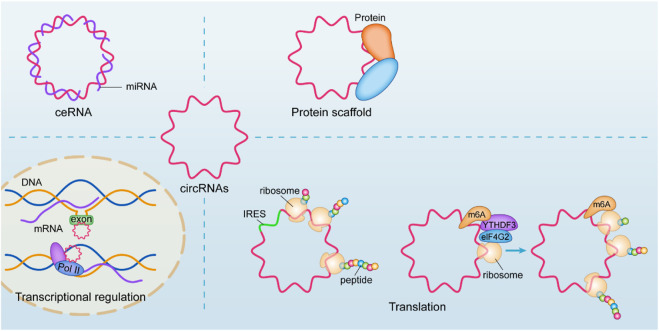
Biological functions of circRNAs. An increasing number of studies have shown that its mechanisms and physiological functions are diverse, such as regulating transcription and binding with its host gene to form an R-loop structure and upregulating the transcription process of skipping exons or intercepts, cooperating with the U1 snRNP junction for Pol II regulation of nuclear transcription. CircRNA with IRES structure can be used as a template for translation. More studies have also found that its recognition by YTHDF3 after m6A methylation, which recruits eIF4G2, can also enable circRNA translation. CircRNAs can also affect protein function by interacting with DNA or RNA-binding proteins or affect protein-to-protein interactions.

In terms of mechanism, the most common studies have focused on its molecular sponge as a microRNA, competitively inhibiting its host gene by affecting its intervention transcription function, also known as the competing endogenous RNAs (ceRNA) mechanism (Panda, 2018). An increasing number of studies have shown that its mechanisms and physiological functions are diverse (Chen, 2020a), such as regulating transcription and binding with its host gene to form an R-loop structure and upregulating the transcription process of skipping exons or intercepts (Conn et al., 2017), cooperating with the U1 snRNP junction for Pol II regulation of nuclear transcription (Li et al., 2015). The translation function of circRNA has received increasing attention in recent years, and circRNA with IRES structure can be used as a template for translation, translating the corresponding biologically functional peptide segment (Legnini et al., 2017). Moreover, circRNAs with ORFs are translated in a rolling circle manner, even up to a hundredfold linear translation, due to the property of their circularity (Abe et al., 2013). However, more studies have also found that its recognition by YTHDF3 after N6-methyladenosine (m6A) methylation, which recruits eIF4G2, can also enable circRNA translation (Di Timoteo et al., 2020). CircRNAs can also affect protein function by interacting with DNA or RNA-binding proteins (Du et al., 2017; Luo et al., 2019; Zhou et al., 2020a; Huang et al., 2020) or affect protein-to-protein interactions (Zhou et al., 2020a). (Figure 1)

The metabolic mechanisms of circRNAs have also been clarified in recent years, including their upstream regulation and downstream metabolism (Xiao et al., 2020). CircRNAs are regulated by cis-acting elements and transcription factors (Ashwal-Fluss et al., 2014). The reverse repeated Alu sequence in the flank inclusion promotes exon circularization (Zhang et al., 2014). According to recent literature, N6-methyladenosine (m6A) controls the biogenesis of circRNA. Methyltransferase-like 3 (METTL3) or YTH domain 1 (YTHDC1) is reported to regulate circRNA (Di Timoteo et al., 2020). It has also been reported to be regulated by m6A concerning its transport out of the nucleus and degradation, methylated circRNA translocated into the cytosol through YTHDC1 recognition (Chen et al., 2019a), and its degradation through YTHDF2 (Park et al., 2019). Other degradation mechanisms, including RNase L, have also been reported to degrade circRNAs (Liu et al., 2019).

The physiopathological functions of circRNAs have been elucidated in a significant fraction of diseases as regulatory roles, involving almost all physiological processes in all organisms and regulating the function of cells and organs. Its clinical transformation has also been documented, and circRNAs can be used as biomarkers of liquid biopsies for the early diagnosis of diseases such as tumors and the evaluation of disease progression (Li et al., 2021a; Li et al., 2021b; Gui et al., 2021; Kuo et al., 2021; Zhang et al., 2022a; Kristensen et al., 2022).

## 3 CircRNAs in OA chondrocytes

Cartilage degeneration is a significant event in the development and progression of OA, and in this study, we summarize the various pathological processes in which circRNAs participate in cartilage degeneration.

### 3.1 CircRNAs in cartilage development and differentiation

CircRNAs have significantly different expression levels at different stages of developmental processes and promote the differentiation of bone marrow-derived stem cells or adipose-derived stem cells (Zhou et al., 2021a). In recent years, research on OA has also focused on bone and cartilage development and differentiation. In terms of noncoding RNAs, it has been well documented that miRNAs act as important regulators involved in cartilage development and differentiation. Especially in recent years, there has been a blowout increase in the study of miRNAs in cartilage differentiation, and many miRNAs have been identified to play a regulatory role in cartilage differentiation. miRNAs can also regulate mesenchymal stem cell differentiation by targeting the transcription of growth-related genes such as IHH, SOX5/6, and Sox9. (Iaquinta et al., 2021).

As a result, circRNAs, as noncoding RNAs closely related to miRNAs, are also likely to play a regulatory role in the development and differentiation of cartilage. CircPSM3 has been proven to regulate cartilage differentiation in cartilage and is upregulated in OA, targeting miRNA-296-5p. After detecting BMP2, BMP4, BMP6, and Runx2 at the mRNA and protein levels, it was found that high expression of miRNA-296-5p could effectively promote OA chondrocyte differentiation, while a miRNA-296-5p inhibitor could reverse the differentiation of OA chondrocytes promoted by si-circPSM3 (Ni et al., 2020). The recent study also showed that the circATRNL1/Sox9 pathway presented a clear high expression and a positive correlation with increased chondrogenesis in adipose mesenchymal stem cells and was regulated by miR-145-5p (Zhu et al., 2021a). CircNFIX/miR758-3p/KDM6A axis has also been reported as a possible target for regulating chondrogenesis (Liao et al., 2022). Furthermore, differentiation into proliferative chondrocytes may effectively increase the likelihood of cartilage repair, and studies on the roles of circRNAs in chondrogenesis may be necessary.

### 3.2 circRNA is involved in cartilage degeneration

**FIGURE 2:**
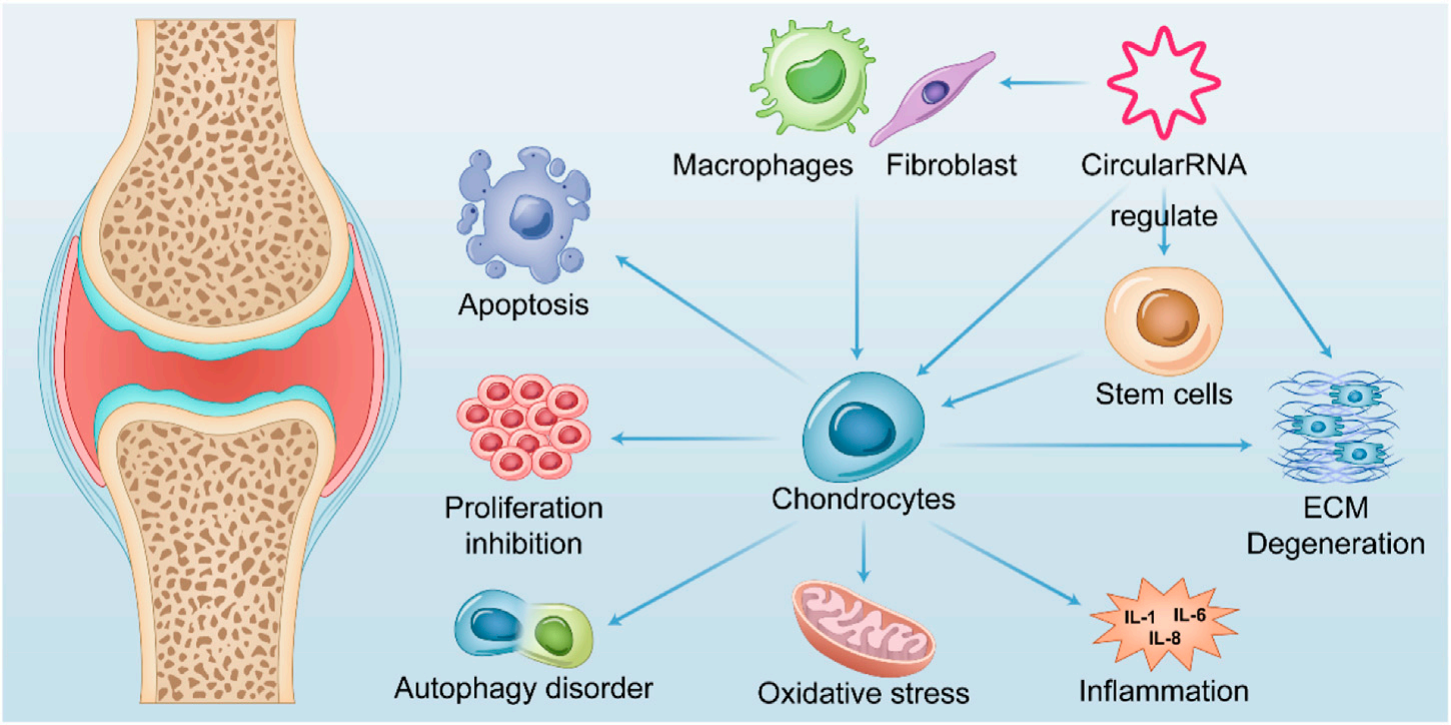
Summary of the role of CircRNAs in OA. CircularRNAs function in multiple tissues within the OA joint and their upregulation or downregulation in chondrocytes is associated with apoptosis, abnormal autophagy, impaired proliferation, oxidative stress, and cellular inflammatory status of chondrocytes, thereby mediating catabolism of extracellular matrix. Further exacerbates chondrocyte degeneration after losing the extracellular matrix environment. It also mediates fibroblast proliferation and polarization of macrophages in synoviocytes, leading to their release of inflammatory factors that exacerbate chondrocyte degeneration.

Cartilage degeneration and loss as typical osteoarthritis and explicit pathophysiological changes are most studied in osteoarthritis research. Many studies have shown that circRNAs are involved in a variety of microRNA-regulated cartilage loss through ceRNA mechanisms, including apoptosis, proliferative function change, autophagic function change, inflammatory status, and degradation of the extracellular matrix of chondrocytes (Mao et al., 2021a). Although a considerable number of current studies confirm it, most of them appear too similar, making it quite difficult and necessary to identify critical circRNAs and pathways with regulatory functions. Nevertheless, an increasing number of studies have verified multifaceted functions, such as validating the simultaneous regulation of cartilage inflammation, chondrocyte apoptosis, and cartilage extracellular matrix degradation by circRNAs, which reflects that inflammation, apoptosis, and extracellular matrix degradation may be common in the terminal state of OA cartilage (Figure 2).

In this review, we summarize the existing research on the regulatory mechanisms of circRNAs in OA cartilage and present the results in Table 1. The mechanisms, pathways, biological functions, and animal models used in the research of circRNAs in OA progression are summarized. Considering that these studies all experimentally validated circRNAs and their targets for expression levels at the nucleic acid or protein level, we pooled and analyzed these circRNAs and their targets. We found that 42 circRNAs were downregulated and 61 upregulated in OA cartilage. After performing an analysis of circRNAs differentially expressed in OA, we found that the CircRNAs play a significant role in OA mainly through the following pathways and genes, including the classic Ras/MAPK pathway, PI3K/AKT pathway, TGF-β/SMAD path, JAK/STAT path, and FGF/ERK signal path. In addition, circRNAs in OA regulate SOX9 related cartilage differentiation pathway, BMPR2 represented osteoblastic differentiation pathway, CCDN1 represented cell cycle pathway, PON2 related oxidative stress pathway or directly regulate genes related to extracellular matrix metabolism such as TIMP3, MMP13 and ADAMTS5 related matrix metalloproteinase. Meanwhile, circRNAs also participate in the regulation of the expression of a considerable number of transcription factors like FOXO1 and KLF5 as well as ubiquitination related genes like FBXO21 and FBXW7, to affect the expression of downstream genes (Table 1).

**TABLE 1:** CircRNAs in OA progression.

circRNA	Mechanism	Pathway	Biological function regulated by circRNA	Expression	Animal model	Methodes to knockdown/overexpression	References
circ_0114876	ceRNA	miR-1227-3p/ADAM10	Apoptosis, proliferation,Inflammation and ECM Degradation	up			Ou et al. (2022)
Circ_0136474	ceRNA	miR-140-3p/MECP2	ECM degradation, apoptosis, and inflammation	up			Cheng et al. (2022)
CircFOXO3	RNA-protein binding	FOXO3/PI3K/AKT	autophagy	down	DMM(mice)	IA-injection (lentivirus)	Zhao et al. (2022)
hsa_circ_00046621	ceRNA	miR-424-5p/VEGFA	Apoptosis, proliferation, and ECM Degradation	up			Xie et al. (2022)
circRNA-MSR	ceRNA	miR-761/FBXO21	autophagy	up			Jia et al. (2022)
circ_0008365	ceRNA	miR-338-3p/SOX9	apoptosis and ECM degradation	down			Shuai et al. (2022)
circCREBBP	ceRNA	miR-1208/TGFβ2/ALK1/Smad1/5	apoptosis, inflammation and ECM degeneration	up	DMM(mice)	IA-injection (AAV)	Xu et al. (2022)
Circ_0022383	ceRNA	miR-3619-5p/SIRT1	apoptosis, inflammation and ECM degeneration	down			Qian et al. (2022)
CircKMT2E	ceRNA	miR-140-5p/TLR4	apoptosis	up			He et al. (2022a)
Circ_0037658	ceRNA	miR-665/ADAMTS5	Apoptosis, proliferation,Inflammation and ECM Degradation	up			Li et al. (2022b)
CircPRKCH	ceRNA	miR-145/HGF	apoptosis and ECM degradation	up	monosodium iodoacetate	IA-injection (lentivirus)	Que et al. (2022)
CircSCAPER	ceRNA	miR-127-5p/TLR4	apoptosis, ECM degradation, inflammation and oxidative stress	up			Zhang et al. (2022c)
circMELK	ceRNA	miR-497-5p/MYD88/NF- κ B	Apoptosis and Autophagy	up			Zhang et al. (2022d)
CircSPI1_005	ceRNA	miR-370-3p/MAP3K9	Apoptosis, proliferation,Inflammation and ECM Degradation	down	DMM(mice)	IA-injection (AAV)	Zhou et al. (2022)
circ_0128846	ceRNA	miR-940/PTPN12	Apoptosis, proliferation,Inflammation and ECM Degradation	up			Fu et al. (2022)
circNFKB1	RNA-protein binding	ENO1/NF-κB	ECM catabolism and anabolism	up	DMM(mice)	IA injections (AV)	Tang et al. (2022a)
Circ-NCX1	ceRNA	miR-133a/SIRT1	apoptosis	down			Liu et al. (2022b)
CircRERE	ceRNA	m6A-modified/miR-195-5p/IRF2BPL/β-catenin	ubiquitination	down	DMM(mice)	IA-injection (AAV)	Liu et al. (2022c)
CircCDK14	ceRNA	miR-1183/KLF5	Apoptosis, proliferation and ECM Degradation	down			Lai et al. (2022)
CircHIPK3	ceRNA	miR-30a-3p/PON2	mitochondrial pathway, apoptosis and ECM degradation	down	DMM(mice)	IA-injection (lentivirus)	Shang et al. (2022)
circPhf21a-Vegfa		Vegfa	proliferation and extracellular matrix synthesis	up			Lin et al. (2022)
circPDE4D	ceRNA	miR-4306/SOX9	apoptosis and matrix degradation	down			Gao et al. (2022)
circ_0136474	ceRNA	miR-665/FGFR1	Proliferation, cell cycle and apoptosis	up			Pan et al. (2022)
circ_0000205	ceRNA	miR-766-3p/ADAMTS5	Apoptosis, proliferation, Inflammation and ECM Degradation	up			Li et al. (2022c)
circ_0000423	ceRNA	miRNA-27b-3p/MMP-13	ECM Degradation	up	ACLT (mice)	IA injections (AAV)	Li et al. (2022d)
circVMA21	ceRNA	miR-495-3p/FBWX7	Apoptosis, proliferation,Inflammation and ECM Degradation	down	DMM(rat)	IA injections (AV)	Li et al. (2022e)
circPRKCH	ceRNA	miR-502-5p/ADAMTS5	Apoptosis, proliferation, Inflammation, migration	up			Liu et al. (2022d)
circADAMTS6	ceRNA	miR-324-5p/PIK3R3	Apoptosis, proliferation, Inflammation and ECM Degradation	down			Shen et al. (2022)
Circ_0043947	ceRNA	miR-671-5p/RTN3	Apoptosis, proliferation, Inflammation and ECM Degradation	up			He et al. (2022b)
Circ_0005526	ceRNA	miR-142-5p/TCF4	Proliferation, apoptosis and inflammation	up			Wahafu et al. (2022)
circRHOT1	ceRNA	miR-142-5p/CCND1	Autophagy, Proliferation	down	ACLT (rat)	IA injection (lentivirus)	Man et al. (2022)
Circ-LRP1B	ceRNA	miR-34a-5p/NRF1	proliferation, apoptosis and oxidative stress	down			Zhang et al. (2022e)
circ_0020014	ceRNA	miR-613/ADAMTS5	Apoptosis, proliferation,Inflammation and ECM Degradation	up			Yu et al. (2022a)
Circ_0110251	ceRNA	miR-3189-3p/SPRY1	apoptosis and ECM degradation	down			Zhang et al. (2022f)
circTRIO	ceRNA	miR-136-5p/NAMPT	Apoptosis, proliferation,Inflammation and ECM Degradation	up	DMM(rat)	IA injections (lentivirus)	Yang et al. (2022)
Circ_0008365	ceRNA	miR-324-5p/BMPR2/NF-κB	Apoptosis, Inflammation and ECM Degradation	down			Zhang et al. (2022g)
circ_Rapgef1	ceRNA	miR-383-3p/NLRP3	Proliferation, apoptosis, inflammation	up	ACLT (MICE)		Yan et al. (2022)
CircTMOD3	ceRNA	miR-27a	apoptosis	up			Yu et al. (2022b)
CircSCAPER	ceRNA	miR-140-3p/EZH2	ECM degradation, proliferation, and apoptosis	up			Luobu et al. (2022)
Circ_0020093	ceRNA	miR-181a-5p/ERG	Inflammation, Apoptosis and ECM Degradation	down			Zhu and Guo (2022)
circCCDC66	ceRNA	miR-3622b-5p/SIRT3	Proliferation, apoptosis, Inflammation	up			Zhang et al. (2022h)
mmu_circ_0001598	ceRNA	miR-127-3p	proliferation, apoptosis, and ECM degradation	up	ACLT (mice)	IA injections (lentivirus)	Apizi et al. (2021)
CircPan3	ceRNA	miR-667-5p/ghrelin	Ghrelin System/autophage	down	IL-1β(rat)		Zeng et al. (2021)
Circ_SPG11	ceRNA	miR-665/GREM1	apoptosis and ECM degradation	up			Ouyang et al. (2021)
circPhc3	ceRNA	microRNA-93-3p/FoxO1	mechanical loading-regulation	down	DMM(mice)		Wang et al. (2022b)
hsa_circ_0005567	ceRNA	miR-492/SOCS2	M2 type macrophage polarization	down			Zhang et al. (2021b)
Circ_0045714	ceRNA	miR-331-3p/PIK3R3	proliferation, apoptosis, ECM degradation	down			Ding et al. (2021)
circ_SEC24A	ceRNA	miR-26b-5p/DNMT3A	proliferation, apoptosis, inflammation, ECM degradation	up			Zhang et al. (2021c)
CircRNA-MSR	ceRNA	miR-643/MAP2K6	proliferation, apoptosis, inflammation, ECM degradation	up			Jia and Wei (2021)
Circ_0128846	ceRNA	miR-140-3p/JAK2	proliferation, apoptosis, inflammation, ECM degradation	up	DMM(rats)	IA injection (lentivirus)	Li et al. (2021d)
Hsa_circ_0134111	ceRNA	miR-224-5p/CCL1	proliferation, apoptosis, inflammation, ECM degradation	up			Zhang et al. (2021d)
circZNF652	NA	PTEN	apoptosis	up			Yuan et al. (2021)
Circ-SPG11	ceRNA	miR-337-3p/ADAMTS5	proliferation, apoptosis, inflammation	up			Liu et al. (2021a)
circFAM160A2	ceRNA	miR-505-3p/SIRT3	Mitochondrial Stabilization/Apoptosis	down	ACLT (mice)	IA injection (lentivirus)	Bao et al. (2021)
CircFADS2	ceRNA	miR-195-5p methylation	apoptosis	down			Zhang et al. (2021e)
CircHYBID	ceRNA	hsa-miR-29b-3p/TGF-β1	hyaluronan metabolism (ECM degeneration)	down			Liao et al. (2021)
circ_0136474	ceRNA	miR-766-3p/DNMT3A	apoptosis, oxidative stress	up			Zhu et al. (2021b)
circ-BRWD1	ceRNA	miR-1277/TRAF6	proliferation, apoptosis, inflammation, ECM degradation	up			Guo et al. (2021)
Circ_0020093	ceRNA	miR-23b/SPRY1	apoptosis, ECM degeneration	down			Feng et al. (2021)
circPDE4B	protein scaffold	RIC8A/MID1	ECM degeneration	down	ACLT (mice)	IA injection (AAV)	Shen et al. (2021)
Hsa_circ_0032131	ceRNA	miR-502-5p/PRDX3/Trx1	cell circle, ECM degeneration, Apoptosis	up	DMM(rats)	IA injection (lentivirus)	Xu and Ma (2021)
circ_0128846	ceRNA	miR-127-5p/NAMPT	apoptosis, inflammation, and ECM degradation	up			Liu et al. (2021b)
CircATRNL1	ceRNA	miR-153-3p/KLF5	inflammatation, apoptosis and ECM degradation	down			Wang et al. (2021a)
CircSLC7A2	ceRNA	load/FUS/CircSLC7A2/miR-4498/TIMP3	ECM degeneration, apoptossis	down	ACLT (mice)	IA injection (AAV)	Ni et al. (2021)
CircTMBIM6	ceRNA	miR-27a/MMP13	ECM degeneration	up			Bai et al. (2020)
Circ_0116061	ceRNA	miR-200b-3p/SMURF2	apoptosis, inflammation,proliferation	up			Zheng et al. (2021)
Circ_0001103	ceRNA	miR-375/SIRT1	ECM degeneration, apoptosis,proliferation	down			Zhang et al. (2021f)
CircSEC24A	ceRNA	miR-142-5p/SOX5	inflammation, apoptosis,proliferation	up			Shi et al. (2021)
Circ_0134111	ceRNA	miR-515-5p/SOCS1	proliferation, apoptosis,inflammation	up			Wu et al. (2021a)
circ-IQGAP1	ceRNA	miR-671-5p/TCF4	inflammation, apoptosis	up			Xi et al. (2021)
hsa_circ_0094742	ceRNA	microRNA-127-5p/latexin	apoptosis, ECM degeneration	down			Sun et al. (2021)
Circ_SLC39A8	ceRNA	miR-591/IRAK3	proliferation, apoptosis,inflammation	up			Yu et al. (2021)
circ-PRKCH	ceRNA	miR-140-3p/ADAM10	proliferation, apoptosis,inflammation	up			Zhao et al. (2021)
CircADAMTS6	ceRNA	miR-431-5p	apoptosis	down			Fu et al. (2021)
Circ0083429	ceRNA	miR-346/SMAD3	ECM degeneration	down	ACLT (mice)	IA injection (AAV)	Yao et al. (2020)
Circ_0114876	ceRNA	miR-671/TRAF2	ECM degeneration, inflammation	up			Wang et al. (2021b)
Circ_0045714	ceRNA	miR-218-5p/HRAS	ECM degeneration, apoptosis,inflammation	down			Jiang et al. (2021c)
circRSU1	ceRNA	miR-93-5p/MAP3K8	oxidative stress regulation	up	DMM(mice)	IA injection (AAV)	Yang et al. (2021)
Circ_DHRS3	ceRNA	miR-183-5p/GREM1	apoptosis and ECM degeneration	up			Jiang et al. (2021d)
CircSERPINE2	ceRNA	miR-495/TGFBR2	apoptosis and ECM degeneration	down			Zhang et al. (2020b)
circANKRD36	ceRNA	miR-599/Casz1	apptosis and inflammation	down			Zhou et al. (2021c)
CircRNA_0092516	ceRNA	miR-337-3p/PTEN	proliferation, apptosis,inflammation	up	DMM(mice)	IA injection (lentivirus)	Huang et al. (2021)
ciRS-7	ceRNA	ciRS-7/miR-7/PI3K/AKT/mTOR	autophage	down			Zhou et al. (2019a)
CircCDH13	ceRNA	miR-296-3p/PTEN	apoptosis, ECM degeneration	up	DMM	IA injection (AAV)	Zhou et al. (2021d)
circPDE4D	ceRNA	miR-103a-3p/FGF18	ECM degeneration	down	DMM(mice)	IA injection (AAV)	Wu et al. (2021b)
Hsa_circ_0005567	ceRNA	miR-495/ATG14	autophage, apoptosis	down			Zhang et al. (2020a)
CircRNA HIPK3	ceRNA	miR-124/SOX8	apoptosis	up			Wu et al. (2020)
circRNF121	ceRNA	LEF1/circRNF121/miR-665/MYD88/NF-Кb	ECM degeneration, apoptosis and proliferation	up	DMM(rats)	IA injection (lentivirus)	Wang et al. (2020b)
CircCDK14	ceRNA	miR-125a-5p/Smad2/TGF-β	ECM degeneration, apoptosis and proliferation	down	DMM(rabbit)	IA injection (AAV)	Shen et al. (2020)
Circular RNA-9119	ceRNA	microRNA-26a/PTEN	apoptosis	down			Chen et al. (2020a)
CircVCAN	NA	NF-κB	cell circle, apoptosis	up			Ma et al. (2021)
CircRNA-UBE2G1	ceRNA	miR-373/HIF-1a	apoptosis, proliferation	up			Chen et al. (2020b)
CircPSM3	ceRNA	miRNA-296-5p	differentiation, proliferation	up			Ni et al. (2020)
Circular RNA-CDR1as	ceRNA	microRNA-641/FGF-2/MEK/ERK	ECM degeneration, inflammation	up			Zhang et al. (2020c)
Circ_0136474	ceRNA	miR-127-5p/MMP13	Inflammation, proliferation	up			Li et al. (2019)
CircSERPINE2	ceRNA	miR-1271/ERG	ECM degeneration, apoptosis	down	ACLT (rabbit)	IA injection (AAV)	Shen et al. (2019)
ciRS-7	ceRNA	ciRS-7/miR-7	proliferation, apoptosis, inflammation	down			Zhou et al. (2019a)
circRNA.33186	ceRNA	miR-127-5p/MMP-13	apoptosis	up	DMM		Zhou et al. (2019b)
Circular RNA Atp9b	ceRNA	miR-138-5p	ECM degeneration, inflammation	up			Zhou et al. (2018)
Hsa_circ_0045714	ceRNA	miR-193b/IGF1R	proliferation, apoptosis and ECM degeneration	down			Li et al. (2017b)
hsa_circ_0005105	ceRNA	miR-26a/NAMPT	ECM degeneration, inflammation	up			Wu et al. (2017)
circRNA-CER	ceRNA	MiR-136/MMP13	ECM degeneration	up			Liu et al. (2016)

ECM, extracellular matrixc; ceRNA, competing endogenous RNAs; DMM, medial meniscal enucleation models; ACLT, anterior cruciate ligament transection; IA injection, intra-articular injection; AAV, adeno-associated virus; AV, adenovirus.

#### 3.2.1 Regulation of chondrocyte proliferation, apoptosis, and autophagy

In studies of cartilage degeneration loss, the status of chondrocytes is undoubtedly crucial. Involvement of circRNAs has been found in apoptotic pathways, changes in chondrocyte proliferation function, and changes in autophagy function (Jiang et al., 2020). A large number of studies have shown that circRNAs play a regulatory role in the apoptosis of chondrocytes (Table 1).

Almost all of the existing studies focus on the ceRNA mechanism. CircRNA regulates the expression of target genes through the competitive binding of miRNA, thus regulating the apoptosis of chondrocytes. Therefore, chondrocyte apoptosis and the weakening of proliferation should be considered the final outcome of cell fate. Targets for therapy should focus on more upstream pathways.

Reasonable autophagy often protects joint chondrocytes from further damage (Valenti et al., 2021). In osteoarthritic cartilage, however, chondrocyte autophagy disorders are also a significant cause of cartilage degeneration. CircRNAs significantly regulate autophagy in chondrocytes, and the ciRS-7/miR-7/PI3K/AKT/mTOR pathway (Zhou et al., 2020b), Hsa_circ_0005567/miR-495/ATG14 pathway (Zhang et al., 2020a), circRNA-MSR/miR-761/FBXO21 (Jia et al., 2022)and CircPan3/miR-667-5p/ghrelin (Zeng et al., 2021) have been shown to have regulatory effects in OA.

#### 3.2.2 CircRNAs regulate the inflammatory state of chondrocytes

Osteoarthritis is an inflammatory disease. The inflammatory state of chondrocytes is naturally also considered in the study, and circRNA-mediated inflammatory processes also play an essential role. CircRNAs have a regulatory role in several inflammatory processes (Saaoud et al., 2021). In osteoarthritis models, upregulated or downregulated circRNAs have also been observed to impact the production and degradation of inflammatory factors. The main inflammatory factors regulated by circRNAs were IL-6/IL-8/TNF-α/IL-17, and IL-1β and TNF-α also induced most osteoarthritis *in vitro* cell models. In addition, macrophages within the joint environment have also been implicated in the inflammation of OA cartilage. CircRNAs have also been found to play a regulatory role in inducing macrophage polarization. One study showed that the expression of hsa_circ_0005567 in OA synovium is downregulated. Overexpression of hsa_circ_0005567 inhibits M1-type macrophage polarization and promotes M2-type macrophage polarization. After being treated with the supernatant of LPS-induced THP-1 macrophages, the proliferation of chondrocytes was significantly reduced, while the apoptosis rate was significantly increased. Hsa_circ_0005567 overexpression reversed this phenomenon. Mechanistically, hsa_circ_0005567 acts through the miR-492/SOCS2 axis to suppress M1 macrophage polarization and thereby mitigate chondrocyte apoptosis in OA cartilage (Zhang et al., 2021b). A large number of studies have shown that circRNAs play a regulatory role in the inflammation of chondrocytes (Table 1). These studies demonstrate that circRNAs can influence the fate of chondrocytes and their extracellular matrix through the involvement of miRNAs in the regulation of inflammatory factors.

#### 3.2.3 Changes in the extracellular matrix of chondrocytes

Degradation of the extracellular matrix is an essential mechanism for the development of osteoarthritis and is also thought to be a characteristic phenotype of osteoarthritis. MMP1, MMP3, MMP13, aggrecanase, ADAMTS4, ADAMTS5, and cathepsins have been proven to be specific markers of matrix degradation. Currently, many studies have confirmed that circRNAs, by competitively inhibiting miRNAs, affect the function of their target genes, causing the composition of the extracellular mechanism to change, eventually leading to the occurrence and progression of osteoarthritis.

#### 3.2.4 circRNA is involved in the oxidation stress process

Oxidative stress regulated by circRNAs has also been observed to be involved in several other mechanisms that cause damage to chondrocytes. YANG Y et al. found that circRSU1 in human chondrocytes affects downstream oxidative stress processes in osteoarthritis models through the miR-93-5p/MAP3K8 axis. Abnormally high expression of circRSU1 leads to the production of more ROS and the loss of cartilage extracellular matrix, leading to the occurrence and development of osteoarthritis (Yang et al., 2021). The role of circRNA in oxidative stress deserves attention, and Zhao found that in NASH (nonalcoholic fatty liver disease), mitochondrial circRNA-circSCAR reduces mitochondrial oxidative stress processes affecting the interaction between CypD and ATP5B. In the case of lipid exposure, circSCAR is regulated by endothelial mesh stress and PCG-1. After overexpression of circSCAR in mitochondria through mitochondrial-targeted nanograms in mice, oxidative stress in the NASH liver was significantly reversed, and liver function was improved (Zhao et al., 2020). This study reveals that circRNAs also play an essential regulatory role in metabolic diseases and mitochondrial function. At the same time, a significant proportion of patients with OA have significant metabolic syndrome, considering the possibility of metabolic osteoarthritis (Kuusalo et al., 2021). On the other hand, mitochondria also play an essential role in aging-induced degenerative diseases such as OA (Sun et al., 2020). CircRNAs have also been reported to play an essential role in mitochondrial stabilization in OA. SIRT3, an essential gene for mitochondrial function, significantly decreased expression in OA chondrocytes and was regulated by miR-505-3p. Overexpression of miR-505-3p can cause ROS to rise, and apoptosis of chondrocytes increases, while overexpression of CircFAM160A2 plays a therapeutic role. Both *in vivo* and *in vitro* experiments have demonstrated that CircFAM160A2 regulates chondrocyte mitochondrial stability and chondrocyte apoptosis through the miR-505-3p/SIRT3 axis (Bao et al., 2021). Recent studies have also demonstrated that CircHIPK3, through the mir-30a-3p/PON2 axis, regulates mitochondrial function, which in turn affects chondrocyte apoptosis (Shang et al., 2022). These findings suggest that our circRNA may also regulate mitochondria in OA, providing new research ideas.

#### 3.2.5 circRNAs act as protein scaffolds in OA chondrocytes

It is clear that circRNAs’ rich mechanisms of action have been increasingly recognized in recent years, such as translation function and interactions with proteins, and in OA studies, little is known about mechanisms other than ceRNA. In a recent study, Shen et al. found that the reduction in circPDE4B in cartilage in OA patients was regulated by upstream FUS (an RNA-binding protein). The downregulation of circPDE4B resulted in the degradation of the extracellular matrix and decreased viability of chondrocytes. At the same time, AGO2 RIP was found to not function through the ceRNA mechanism. After cloning and amplification of its ORF sequence, no protein translated by circPDE4B was identified. Therefore, after RPD-MS and qRT–PCR verification, the authors found that RIC8 guanine-nucleotide exchange factor A (RIC8A) interacted with circPDE4B. Mass spectrometry has identified MID1 as an E3 ligase interacting with circPDE4B. After identifying the downstream pathway, the author screened out the p38/MAPK pathway. In medial meniscal enucleation models (DMM) model mice, overexpressed circPDE4B has also been proven to inhibit activating the RIC8A and p38/MAPK pathways and reverse the OA phenotype in mice, indicating that circPDE4B can be an effective molecular target drug for OA (Shen et al., 2021). Recent studies have shown that circNFKB1 regulates the expression of its host gene NFKB1 by interacting with the ENO1 protein (Tang et al., 2022a). A novel study found that circFOXO3 regulated the downstream PI3K/Akt pathway and affected chondrocyte autophagy through interaction with its parental gene FOXO3 (Zhao et al., 2022). In the future, more circRNA functions and mechanisms of action may be revealed in OA, which also suggests the possibility of circRNA as a molecularly targeted drug.

## 4 CircRNA is involved in the regulation of the environment within the joint

As a multi-component organization, osteoarthritis is a complex mechanism and an unclear chronic inflammatory disease, and changes in the joint environment are also essential in its development. Current studies of osteoarthritis also focus on other components of the joint, including synovium and subchondral bone, and abnormal vascular and neural factors in osteoarthritis (Ching et al., 2021; Zhang and Wen, 2021).

### 4.1 CircRNA is abnormally expressed in the synovium or infrapatellar fat pad of patients with osteoarthritis

The role of the synovium is not negligible in the development of osteoarthritis. Synovitis is often observed in the OA joint. Both synovial hyperplasia and the secretion of proinflammatory factors drive the progression of osteoarthritis (van den Bosch et al., 2020). MRI and ultrasound have identified a positive correlation between the risk of structural progression of synovial and osteoarthritis and joint symptoms (Roemer et al., 2011). Shuai et al. performed circRNA sequencing of OA synovial samples and controls and identified 122 circRNAs differentially expressed in osteoarthritic synovium. Using GO analysis and KEGG enrichment analysis, differentially expressed circRNAs were rich in adhesion molecules, tumor pathways, TGF-β, and some osteoarthritis pathways, such as Hippo and WNT pathways. This article also establishes the circRNA-miRNA network, exploring possible molecular regulation mechanisms for specific expression of circRNAs, and the miR-20, miR-29, and miR-136 families have all been reported in previous OA studies and interact with several differentially expressed circRNAs (Xiang et al., 2019). On the other hand, the infrapatellar fat pad and the surrounding synovium are also essential tissues involved in regulating the intra-articular environment. Circular RNA expression profiles of the infrapatellar fat pad/synovium unit reveal that hsa_circ_0005265 was down-expressed in both OA synovium and IPFP, targeting hsa-miR-6769b-5p and hsa-miR-1249-5p (Jiang et al., 2021a). However, the molecular mechanisms of differentially expressed circRNAs in OA synovium or infrapatellar fat pad in the progression of OA have not been thoroughly investigate.

### 4.2 Abnormal expression of circRNA in osteoclasts of osteoarthritis

Changes in subchondral bone, especially the imbalance between rupture and remodeling, are also essential mechanisms for the development of osteoarthritis (Fan et al., 2021). In such a process, osteoclasts play a central role (Zhu et al., 2020). Dou C and others first identified the differential expression of circRNAs in abnormally activated osteoclasts. Many differentially expressed circRNAs were identified in osteoclasts and activated osteoclasts, suggesting that circRNA may also be extensively involved in remodeling subchondral bone. In another study, the expression of circRNA in bone marrow stromal cells during RANKL- and CSF1-stimulated osteoclast formation was sequenced, and their difference was analysed. This study focuses on the role of circRNA-28313 in bone marrow osteoclast differentiation. The results showed that circRNA-28313 knockout inhibited the differentiation of bone marrow mesenchymal stem cells and RANKL-induced osteoclasts and partially prevented the bone loss induced by ovariectomy (OVX). Further downstream experiments further found that circRNA-28313 alleviated miR-195a-mediated inhibition of CSF1 through a ceRNA mechanism, thereby regulating osteoclast differentiation (Chen et al., 2019b). The destruction of subchondral bone in OA is related to bone destruction between OA and osteoporosis, and RANKL was also identified in OA (Kovacs et al., 2019). These studies also indicate that circRNA is more likely to participate in the pathogenesis of osteoarthritis.

### 4.3 Abnormal expression of circRNA in osteoarthritis meniscus

Meniscal degeneration and wear are also prevalent in the knee tissue of OA patients, and a large number of OA model animals have used medial meniscal enucleation models to mimic OA (Zaki et al., 2021). Few studies have focused on the mechanism of meniscal changes in OA, but the meniscus plays an essential role in the stability of joints and the protection of articular cartilage in the function of the knee joint. Bin Wang et al. performed bioinformatics analysis and prediction using public databases (GEOs) and revealed 360 differentially expressed genes in the OA meniscus. It is expected that hsa_circ_0025119, hsa_circ_0025113, hsa_circ_0009897, and hsa_circ_0002447 are the most critical circRNAs. The article failed to verify and identify the expression and function of circRNA in subsequent experiments, it also suggested that circRNA might also play a regulatory role in the OA meniscus (Wang et al., 2020a). Meanwhile, The hsa_ circ_ 0018069/mir-147b-3p/tjp2 axis was also found to play a regulatory role in the OA meniscus (Jiang et al., 2021b).

## 5 Upstream regulation and downstream metabolism of circRNAs in OA

The reason for the change in circRNA in OA is nothing more than the increase and decrease in metabolism. The metabolism and upstream regulation of circRNAs have also been hot topics of research, with clear evidence that circRNAs are regulated by transcription factors. In OA studies, there has not been much research on the upstream regulatory factors of circRNAs in osteoarthritis. Wang et al. found that LEF1, as a transcription regulator, affects the expression changes of downstream circRNF121, regulates the miR-665/MYD88/NF-Кb pathway, and ultimately regulates the apoptosis and proliferation of chondrocytes and the metabolism of extracellular matrix (Wang et al., 2020b). RNA binding proteins regulate back splicing mainly by directly bridging distal splice sites and by binding to intronic complementary sequence (ICS) (Chen, 2020a). RNA binding proteins including QKI, HNRNPL, Mbl, SLU7, NF110, NF110, DHX9, ADAR1 were reported to potentially regulate back splicing of circRNAs (Ashwal-Fluss et al., 2014; Conn et al., 2015; Ivanov et al., 2015; Aktas et al., 2017; Li et al., 2017a). Some of them, such as QKI and DHX9, were reported to be differentially expressed in osteoarthritic tissues as well (Li et al., 2016; Tang et al., 2022b), which may also be one of the upstream mechanisms regulating expression of circRNAs in osteoarthritis. However, another possible change in the expression of circRNA in OA is its downstream metabolic changes. Changes in downstream metabolic clearance have rarely been mentioned in OA studies. With a further understanding of the circRNA metabolic pathway, this may be one of the follow-up research directions.

## 6 The role of circRNA in OA of different etiologies and stages

### 6.1 circRNAs in different subtypes of OA

In fact, from a clinical point of view, some risk factors for osteoarthritis should also be taken into account. OA is a highly heterogeneous disease, and different drivers tend to shape different OA phenotypes (Van Spil et al., 2019). In existing circRNA-related studies, different types of osteoarthritis caused by different factors have not yet been considered by most researchers. However, some factors were considered, such as the specimen selection of load-bearing stress areas in osteoarthritis cartilage compared with non-load-bearing stress areas. Some studies selected chondrocytes from load-bearing *versus* non-load-bearing areas to identify differential expression. The etiology of osteoarthritis in the clinic is diverse and complex, and it has been established as a risk factor for several conditions: obesity, physical activity, structural factors, and genetics (Martel-Pelletier et al., 2016). Patients with OA of different etiologies or etiological factors should be treated separately. Therefore, studies should be more refined regarding OA of different etiologies, and corresponding studies aimed at different etiological subtypes of OA, such as RNA sequencing and identification of differential expression in patients with distinct metabolic profiles or those with distinct stress factors, may also yield different results. The construction of different OA models usually simulates different OA initiating factors, especially the design of animal models (Zaki et al., 2021). Most research on OA circRNAs is anterior cruciate ligament transection (ACLT) and medial meniscal enucleation models (DMM) models in rats and mice. These two models can simulate the initiating factors of traumatic osteoarthritis. More pathogenetic factors for OA should be considered. Designing different animal models may be a solution to this problem. Additionally, molecular subtype of OA has been mentioned by more and more researchers. Julia Steinberg et al. conducted a cluster analysis of mRNA sequencing data obtained from cartilage and synovium samples of OA patients. They discovered two subgroups in the synovium, one related to inflammation and the other related to extracellular matrix metabolism. High-grade inflammation of cartilage was also linked to female gender and proton pump inhibitor use (Steinberg et al., 2021). In another study, researchers from China sequenced cartilage samples from 131 OA patients and carried out a cluster analysis of their expression profiles. The patients were divided into four subtypes: a subtype characterized by glycosaminoglycan metabolism disorder, a subtype marked by collagen metabolism disorder, a subtype with sensory neuron activation, and an inflammation subtype (Yuan et al., 2020). Biochemical markers have also been employed to cluster and classify patients into three groups, C1, C2, and C3. C1 is associated with low tissue turnover, including low repair and turnover of articular cartilage and subchondral bone. C2 is characterized by structural damage, such as high bone formation and resorption and cartilage degeneration. C3 is linked to systemic inflammation, joint tissue degeneration, and cartilage degeneration. In the FNIH/OAI cohort, C1 had the highest proportion of progressors, C2 was linked to the progression of mechanical structure, and C3 was associated with pain, which was consistent with molecular typing (Angelini et al., 2022). Future research can construct circRNA expression profiles of patients with different OA subtypes and conduct in-depth exploration of the relevant mechanisms.

### 6.2 circRNAs in different stages of OA

Another important aspect is that OA, as a chronic disease, has a significantly long pathological process, and different stages of OA have different characteristics. Sun et al. (2011) measured the miRNA expression profiles of rat articular cartilage at different developmental stages and sequenced femoral head cartilage on zeroth days, twenty-first days, and forty-second days after birth. The authors observed in the results that, on the one hand, some miRNA clusters were continuously expressed at three stages, which may illustrate the existence of specific conserved sequences of miRNAs during development. On the other hand, the expression of some miRNAs changed obviously at different stages, and in the sequencing results combined with PCR validation, it could be found that some miRNAs showed early high expression. In contrast, some showed high expression at later stages of developmen. This study reveals the phased change in the miRNA expression profile at different stages of development. S. A. Ali et al. classified 91 OA patients into early OA (n = 41) and late OA groups (n = 50) using K-L grading of standard X-ray, performed next-generation sequencing on plasma samples from patients, and identified that hsa-mir-335-3p, hsa-mir-199a-5p, hsa-mir-671-3p, hsa-mir-1260b, hsa-mir-191-3p, hsa-mir-335-5p, and hsa-mir-543 were more highly expressed in early OA compared to late OA, while hsa-mir-193b-5p, hsa-mir-193a-5p, and hsa-mir-455-5p were more highly expressed in late OA. Meanwhile, the authors also identified some novel miRNAs, some of which showed high expression in early OA, some of which showed high expression in late OA, and others that showed high expression in both early and advanced stages (Ali et al., 2020). This result illustrated that the expression of miRNAs might change continuously with the progression of OA. Therefore, we can infer that in the different stages of the disease, such as early and late OA, the characteristic circRNA expression profile in the determination stage may bring different results. Determination of circRNA expression profiles based on the patient’s symptoms, signs, functional assessment such as WOMAC or KOOS score of the knee (Roos and Toksvig-Larsen, 2003), and imaging grading such as Kellgren-Lawrence grade to stratify different patients may be considered, which may help us to understand the role of circRNAs in OA more deeply.

## 7 Clinical application and potential of circRNA

### 7.1 circRNA is the marker of a liquid biopsy

CircRNAs are less susceptible to degradation by RNase R due to their lack of 3′and 5′ends, which confers a longer half-life and stability than other noncoding RNAs or mRNAs, suggesting the possibility of circRNAs as indicators for clinical testing. CircRNA as a liquid biopsy indicator has been frequently mentioned in tumor studies (Li et al., 2022a; Wang et al., 2022a; Ruan et al., 2022; Xue et al., 2022). CircRNA was detected in cartilage, synovial fluid, and serum in OA patients and was different from the control group, suggesting that it is an indicator of early diagnosis of osteoarthritis. Chen C et al. measured hsa_circ_101178 levels in serum and fluid and control group serum and fluid in OA patients and found that group OA was significantly higher than the control group. Meanwhile, there was a positive correlation between circ-101178 levels in serum and synovial fluid. In addition, serum hsa_circ_101178 was positively correlated with OA’s KL score and WOMAC pain score (Chen, 2020b). Fangyu et al. identified five circRNAs in the synovial fluid of OA patients who were significantly elevated compared with healthy controls. AUC analysis of diagnostic value found that hsa_circ_0104873, hsa_circ_0104595, and hsa_circ_0101251 can effectively distinguish between OA patients and healthy controls. However, it was also found that three circRNAs were positively correlated with the radiological grade and symptom severity of OA patients (Yu et al., 2018). Similarly, Ying Wang et al. also identified that circ-0032131 levels in the peripheral blood of OA patients were significantly different from those in the healthy population (Wang et al., 2019). Additional studies have also reported that plasma circRNA-016901 can effectively distinguish osteoarthritis from rheumatoid arthritis and is correlated with disease severity (Du et al., 2022). Although these studies failed to carry out clinical trials to prove the diagnostic value of circRNA, they also suggested, to some extent, the possibility of circRNA as a molecular marker for the diagnosis of osteoarthritis.

### 7.2 Clinical transformation potential of circRNA

**FIGURE 3:**
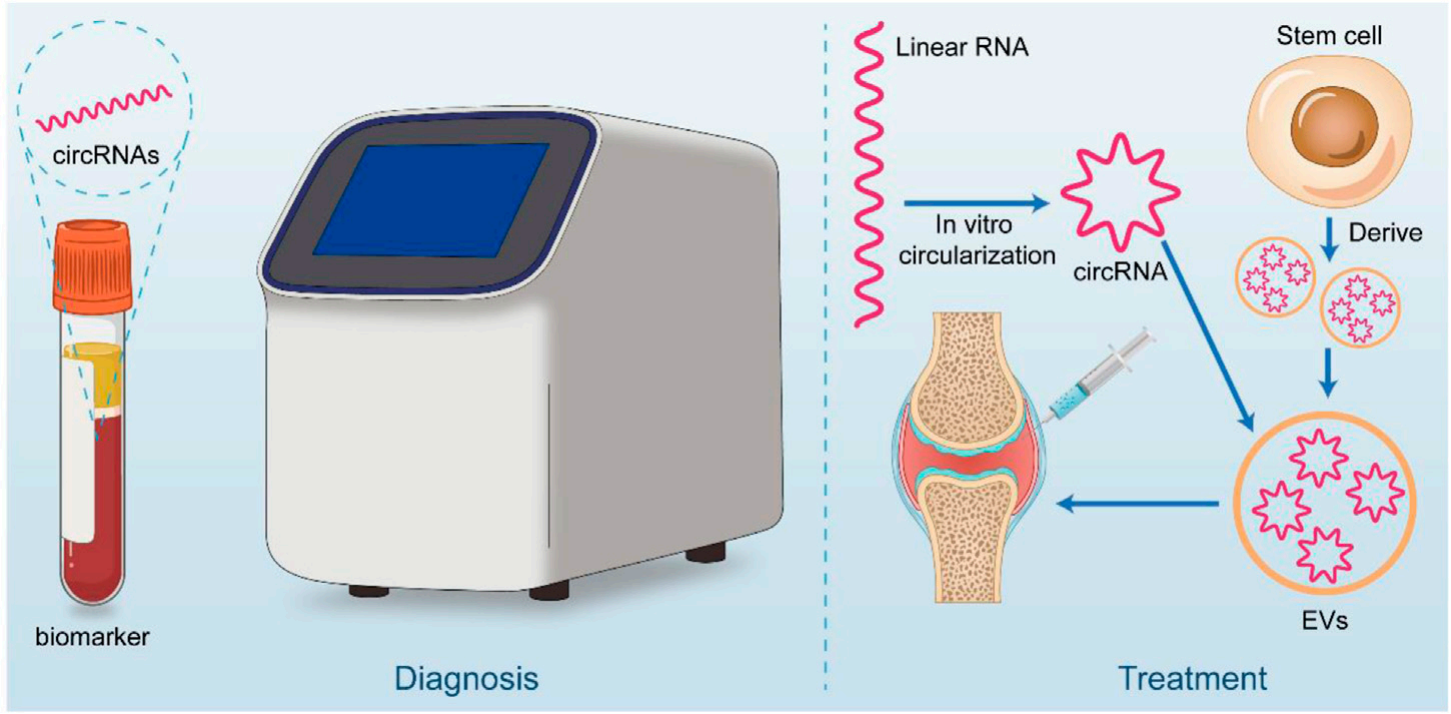
Clinical application potential of circRNA. CircRNA has great potential for both diagnostic and therapeutic applications. In diagnosis, it can be used as a liquid biopsy tool for early screening of OA patients. On the therapeutic side, circRNA is considered to be good for treatment by *in vitro* synthesis (linear RNA cyclization) or stem cell-derived circRNA piggybacked by materials such as extracellular vesicles and injected through the joint cavity.

The study of circRNAs is constantly moving from basic research to clinical translation, and its clinical potential has been explored as it has become more aware of its mechanisms and functions. For example, several animal trials have demonstrated that intraarticular injection of AVV or AV carried shRNA or plasmid targeting circRNAs, and silencing or overexpressing osteoarthritis-associated circRNAs in articular cartilage can effectively alleviate the progression of osteoarthritis in animal models. Treatment with intra-articular injection of sodium hyaluronate and glucocorticoids is widely used in the clinic (Yuan et al., 2022). However, it remains controversial. The development of precision medicine approaches targeting specific nucleic acid drugs within OA joints, combined with individual sequencing results, may lead to better outcomes and personalized treatment options. While circRNAs are stable and are not easily degraded by RNase, the potential for translation and interaction with proteins may make them suitable carriers for nucleic acid drugs (Figure 3).

#### 7.2.1 Extracellular vesicles loaded with circRNA

Extracellular vesicles are also a significant research hotspot, and extracellular vesicles have been suggested to be promising drug carriers, particularly nucleic acid drugs. Extracellular vesicles (EVs) are small vesicles released from different cells into the extracellular matrix, classified by origin and size, and include three subtypes: 1. Apoptotic bodies (500 nm—5 μm); 2. Microvesicles (150–500 nm); 3. Exosomes (40–150 nm), which can participate in intercellular communication (Wu et al., 2022). Exosomes are a subset of EVs secreted by most cells and have good biocompatibility, low toxicity and immunogenicity as well as great designability. They have received extensive attention over the past decades as therapeutic carriers and diagnostic markers. Circular RNAs naturally carried by exosomes have been widely used in the treatment of cancer, cardiovascular, and metabolic diseases (Zhang et al., 2022b).

In the treatment of OA, several studies have proposed that extracellular vesicles may be used as carriers to loading on specific circRNAs and thereby exert their therapeutic effects on OA. As an example, Songlong Li et al. isolated extracellular vesicles from MSCs and observed an increase in COL2A1, Sox9, and aggrecan expression after coculturing circHIPK3-overexpressing extracellular vesicles with chondrocytes, along with inhibited expression of OA-related markers such as MMP-13 and Runx2. Meanwhile, functional experiments also found that CircHIPK3 could alleviate IL-1β-induced inhibition of chondrocyte apoptosis, proliferation, and migration (Li et al., 2021c). Shi Cong Tao et al. found that circRNA3503 was significantly increased after melatonin (MT)-induced cellular sleep. Mechanistically, circRNA3503 acted as a sponge for hsa-mir-181c-3p and hsa-let-7b-3p. Prepared and isolated circRNA3503-loaded extracellular vesicles (circRNA3503-OE-SEVs) from SMSCs. The feasibility of circRNA3503-OE-SEVs in preventing OA progression was validated by *in vivo* and *in vitro* experiments (Tao et al., 2021). In addition, circRNAs transported by exosomes (circ_0001236) suppresses IL-1 β via the miR-3677-3p/Sox9 pathway in TGF-β induced cartilage degeneration, promoting chondrocyte proliferation (Mao et al., 2021b).

#### 7.2.2 mRNA therapeutics

mRNA could theoretically produce any protein on demand inside the cell, enabling treatment of disease. However, linear mRNAs also face many challenges due to their own defects. CircRNAs do not contain a 5′- end cap and a 3'- end PolyA tail and form a covalently closed circular structure by back splicing, protecting circRNAs from degradation by exonucleases. At the same time, circRNAs also need no complex modification when they are synthesized *in vitro*, so circRNAs have the advantages of high stability, low immunogenicity, and long-lasting expression compared to linear mRNA (Santer et al., 2019). Recent studies identified nearly a thousand endogenous circular RNAs that are translatable, half of which can synthesize large molecular weight proteins by rolling circle translation. The authors explore factors that influence translation of circular RNAs and, by optimizing relevant conditions, increase circular RNA protein production several hundred fold, providing a more abundant and long-lasting translated protein product under *in vitro* and *in vivo* conditions (Fan et al., 2022).

*In vitro* synthesis of circRNAs is the basis of drug development, and there have been studies that successfully prepared circRNAs *in vitro* and elucidated their functions. There are currently two major routes known for the *in vitro* synthesis of circular RNAs: direct intramolecular ligation into circles based on catalysis by T4 RNA ligase, and self splicing into circles based on type I intronic ribozymes (T4 bacteriophage or Anabaena) (Chen et al., 2017; Wesselhoeft et al., 2019; Rausch et al., 2021; Qu et al., 2022). There are studies showing that circular RNA synthesized by T4 RNA ligase does not elicit an intracellular innate immune response (Liu et al., 2022a). It provides an important foundation for the further application of circular RNAs synthesized *in vitro* and also holds promising prospects for the further development of nucleic acid aptamers and gene therapy fields based on circular RNA technology. Artificially manufactured circular RNA technology has also been successfully applied in drug development. CircINSR was screened for significant underexpression in heart tissue from patients with heart failure and mice with left ventricular pressure overload induced cardiac remodeling. The authors explored the therapeutic effect using two modalities: AAV loading overexpression plasmid and *in vitro* transcribed CircINSR, and found that *in vitro* transcribed CircINSR could achieve superior therapeutic and protective effects against doxorubicin mediated cardiomyocyte death (Lu et al., 2022).

## 8 Prospect of research

At present, there are a considerable number of studies on the function of circRNAs in osteoarthritis, which fully shows that circRNAs play a regulatory role in OA. However, there are still many deficiencies in the existing research. For example, mechanistic research is limited to acting as a sponge combined with miRNA, which fails to consider the characteristics of diseases, such as OA of different types and stages. However, the potential of circRNAs has been confirmed in many other areas, giving us some directions for studying the role of circRNAs in OA.

Many studies have confirmed the critical role of miRNA in osteoarthritis, and miRNA as a molecular target for precision therapy has also been proven feasible (Ji et al., 2020). CircRNA is an essential competitive inhibitor of miRNA, and its high degree of conservatism and stability makes it possible to fight OA effectively. However, the richness of the mechanism of action of circRNA has been described earlier. The mechanism of circRNA should not be limited to ceRNA. In comparison, only a few articles illustrate its function in OA through interactions with proteins. With the continuous progress of sequencing technology, many studies have shown that circRNAs have the potential for translation and can regulate transcription. Breakthrough progress has been made in the study of circRNA translation products as disease-specific molecular targets in cancer. In triple-negative breast carcinoma, circ-HER2 is expressed in approximately 30% of triple-negative breast carcinoma cells. The translated peptide HER2-103 promotes the proliferation and invasion of triple-negative breast cancer cells, and HER2-103 can also be used as a target molecule for the anti-HER2 targeted drug pertuzumab (Li et al., 2020). In addition, many studies have confirmed the critical role of circRNA translation products in disease development (Zhou et al., 2021b). In addition, a database on circRNA translation has been established. However, up to now, no study of osteoarthritis has found a translational function of circRNA, which may be due to the previous researchers’ lack of in-depth understanding of circRNA, and there are good reasons why we can assume that circRNA in OA may also have the translational function to regulate the progress of the disease.

At present, research on circRNAs in OA has also been performed in a considerable number of experimental animals. As shown in Table 1, animal models used extensively in existing studies are rats, mice, and rabbits. The knockout or overexpression of circRNA was achieved by intra-articular injection of adenovirus (AV), adeno-associated virus (AAV), or lentivirus. However, because of the unique and complex structure of the articular cavity, intra-articular injections can be well absorbed or unknown by articular cartilage and surrounding tissues. Therefore, the effect of such knockout or overexpression in animal models has not been effectively verified, setting obstacles to the development of drug targets and drug safety. All of these findings demonstrate the importance of establishing a circRNA knockout model. Methods to knock out circRNA have always been a challenge. In the past, such knockdowns may have been limited by technical reasons, but the emergence and advancement of new technologies have made it possible to achieve a specific knockout of circRNA (He et al., 2021). Benyu Liu et al. used CRISPR-CAS9 to build circKcnt2 knockout mice to study the role of circRNA in intestinal inflammation. The knockout site was selected as a intronic complementary sequences mediated by flanking introns, which is thought to be the critical sequence for back splicing of circRNAs. circKcnt2 knockout was constructed by deleting the intronic complementary sequences of the genome. Analysis of the expression of circKcnt2 and its host genes in knockout models proved that circKnct2 could be knocked out specifically, while the expression of its host genes was not affected (Liu et al., 2020). The experiment successfully constructed the circRNA knockout model and demonstrated the function of circRNA. Furthermore, a circRNA knockout model was established in OA to improve the current understanding.

In conclusion, circRNA may function as a regulator in OA. A growing body of evidence suggests that circRNAs can regulate chondrocyte proliferation, apoptosis, differentiation, and autophagy, regulate extracellular matrix degradation, and regulate oxidative stress processes and inflammatory processes in chondrocytes. On the other hand, circRNAs can modulate the intra-articular environment, such as the synovium, meniscus, and subchondral bone, and can serve as biomarkers for liquid biopsy. Although the number of studies available is already significant, many deficiencies remain regarding mechanisms, animal model construction, and disease heterogeneity.
